# Facilitating the use of video with teachers of mathematics: learning from staying with the detail

**DOI:** 10.1186/s40594-018-0155-y

**Published:** 2019-01-30

**Authors:** Alf Coles

**Affiliations:** School of Education, University of Bristol, 35 Berkeley Square, Bristol, BS8 1JA UK

**Keywords:** Video, Mathematics teacher learning, Facilitator, Enactivism, Judgement, Observation

## Abstract

**Background:**

This article is focused on the role of the facilitator in the professional development of mathematics teachers. It is a feature of several frameworks for using video that teachers are invited to comment on the detail of what they saw and heard, and base comments on evidence from the video. It is also a common finding that it is hard to establish ‘speaking evidentially’ as a way of working and at the same time, such an expectation is seen to be critical to making discussion productive. This article draws on a paradigmatic case of one video club, during which seven teachers met over a 3-month period, and shared video recordings of their own classrooms. The group of teachers learn how to focus on the detail of video, avoiding judgement, from the first session. The way this is achieved and what it occasions is analysed, within an enactivist methodology.

**Results:**

The analysis of the first meeting of the video club shows how the facilitator has a focus on the *kinds* of comments made by teachers as well as their content. If a teacher makes a contribution that is not of the ‘kind’ required within the way of working, the facilitator is observed to highlight that the participant is not focused on the detail of events, and re-direct the group back to the intended task. This sequence of moves is not captured in current frameworks of how to facilitate video discussion. There is a suggestion that it is the very distinction between observation and interpretation or judgement that is significant for teachers and allows them to re-think their own teaching practices.

**Conclusions:**

This article aims to share awarenesses of the facilitator, concerning how it is possible to focus a group on the detail of events when using video. What the facilitator is doing, in highlighting when a norm has been breached, is making a judgement about the *kind* of comment made by participants, not judging the comment’s content. The meta-focus of the facilitator seems to allow enabling contraints to be placed on discussion while letting conversation follow the interests of participants.

## Background

Teacher J: Because that very first [meeting], I was really judgmental, but once you sort of trained us, it feels really un-inhibiting to watch anyone’s video, you do not think about it.

Teacher P: I was saying, this process has helped me, when I come to compare, when I come to observe now, because I have stopped now thinking about how I would do it and looked at actually what are the children doing, how are they achieving that and what is the teacher doing to get them to achieve that [*extract from audio recording of Meeting 3*].

These teachers were reflecting back on participation in a video club for mathematics teachers (analysed later in this article). Teacher J expresses an awareness of having shifted away from judging what they watch, when observing video recordings and teacher P reflects on no longer considering ‘how I would do it’ when watching someone else teach and rather focusing on what is happening. These teachers mention being ‘trained’ and being aware of a ‘process’ for watching video that seems to have been established from the first session of a video club. In this article, I will be analysing what took place, with the aim of answering the following questions: *how can a facilitator focus discussion away from judgement and onto the detail of events, in the context of professional development using video*? And, *in what ways does focusing on the detail of events occasion subsequent learning for teachers*?

The use of video to support the learning of teachers has been on the increase in the last 10 years, both for pre-service (Brouwer 2011) and in-service teachers ‘in all subject areas, at all grade levels, and all over the world’ (Gaudin and Chalies 2015, p. 41). A Special Issue of the Journal of Mathematics Teacher Education (Karsenty and Sherin 2017) showcased several frameworks for discussing video with teachers of mathematics (Schoenfeld 2017; Karsenty and Arcavi 2017; Hollingsworth and Clarke 2017; Sherin and Dyer 2017), including making use of recent advances in wearable technology. These frameworks come out of sustained interest in the field in using video with teachers for professional development (e.g. Sherin 2007; Star and Strickland 2008; Santagata and Angelici 2010). However, it is only since around 2009 (Elliott et al. 2009) that there has been collective interest in investigating the role of the facilitator of discussion, when working on video with teachers (e.g. in the work of Borko et al. 2011; Coles 2013a; van Es et al. 2014; Zhang et al. 2011). This article contributes to the emerging field exploring the skills needed to facilitate discussion, something that has been considered recently in the attempt to scale up professional development programmes and equip teachers to take on the role of facilitating other teachers (Borko et al. 2014; Jacobs et al. 2017; Lesseig et al. 2017; Schueler and Roesken-Winter 2018).

A common feature of current frameworks for using video is the importance of focusing teachers on the detail of events and avoiding premature evaluation or judgement (e.g. van Es and Sherin 2008). There is an argument, set out in Coles (2014), that if, as a participant, I am allowed to engage in evaluation and judgement (positive or negative) then I will be interpreting what I see in terms of ways of thinking and acting that I already use and my own learning from the video is potentially limited. Lesseig et al. (2017) comment on precisely this feature of facilitating work with video:

a critical factor in promoting productive teacher discussions is the ability to maintain a focus on evidence-based interpretations and avoid premature judgments or evaluation. Evaluation leads to classifying and explaining away events, closing down opportunities for teachers to consider mathematical ideas deeply or reason pedagogically (p. 593).

Different video professional development projects and programmes have different areas of focus, for instance around mathematical thinking or particular content areas (e.g. Sherin and van Es 2009; Borko et al. 2011) but the importance of focusing on evidence from the video cuts across many of them. Having a focus on the detail of events on the video therefore appears important in terms of occasioning the possibility of new insight and learning, but more needs to be elaborated in terms of precisely how and why.

The basis of this article are insights, relevant to the role of the facilitator, from a long-established way using video in the UK that came out of work done at the Open University (OU) (Jaworski 1990). The OU way of working, with an experienced facilitator, is effective at avoiding evaluation in its initial stages and therefore potentially has something to offer to a wide range of ways of working with video. The way of working draws on Mason’s (2002) key distinction between offering ‘accounts of’ phenomena and ‘accounts for’ phenomena. Accounts *of* phenomena aim to report on them in detail, avoiding interpretations, judgments or evaluations. Typically, such accounts will not need to enter into the detail of ergonomic descriptions of movement, for instance, but they do need to point to aspects of phenomena in enough detail that they are observable by others. Accounts *for* phenomena aim to explain what is perceived or interpret it, for example by classifying.

Through looking at one particular video club, I aim to draw out insights around how to establish a focus on the detail of events (accounts of) and offer some suggestive evidence for why this might be important. The data comes from research into the professional learning of teachers of mathematics, funded by the UK’s Economic and Social Research Council (ESRC); this project is offered as a paradigmatic case (Freudenthal 1981, p. 135) of establishing an expectation of discussion beginning with accounts *of* events, avoiding, in the first instance, accounts *for* (which come later). There is a tension, in generating productive discussion with teachers, of needing to somehow direct the focus and yet also needing the teachers themselves to lead, if the conversation is to have relevance to them. In analysing one particular video club, I also aim to bring some theoretical clarity to how a facilitator can meet the dual purposes, which might be seen to be in tension, of directing events and yet following the interests and insights of participants.

In the next section, I review the literature on the role of the facilitator of video use, focusing on what is currently known about generating productive or high-quality discussion. I then introduce the enactivist methodology of the project. Results are presented and analysed, before drawing out implications for working with teachers of mathematics in the context of professional development.

## The role of the facilitator of using video in generating productive discussion

Grossman et al. (2009) challenge teacher educators to develop new approaches to preparing teachers, involving skilled feedback, where ‘practice’ is placed ‘at the centre of all endeavours’ (p. 287). Lampert (2010) invites us to consider the multiple ‘practices’ involved in teaching, rather than separate ‘practice’ from ‘theory’. Of relevance to this article is the broad issue of what might be the theory-practices of teacher educators and, in particular, what are effective theory-practices when facilitating work with mathematics teachers using video? Significant work on this question has been carried out by Borko et al. (2014) and Borko et al. (2017) who have been supporting teachers to take on the role of leading professional development with colleagues. They coined the phrase Mathematics Knowledge for Professional Development (MKPD) to begin to delineate the kinds of knowings that appear necessary for effective facilitation of teacher meetings.

Borko et al. (2014) pointed to work done in classrooms around productive discussions as a starting point for thinking about working with teachers. This was indeed the starting point for Elliott et al. (2009), who drew on two frameworks designed initially for mathematics classrooms in order to develop some theoretical grounding for thinking about the role of a teacher educator. One framework was linked to the development of social discussion norms (Yackel and Cobb 1996) and one focused on the orchestration of discussion (Stein et al. 2008). The social norms framework led Elliott and colleagues to distinguish four features of interaction which were assumed to support teacher learning (translating effective practices from the classroom): (a) sharing, (b) justifying, (c) responding to confusion and errors and (d) questioning. Elliott and colleagues then translated the framework for the orchestration of discussion, from the work of Stein et al. (2008), into five practices suggested as significant for a facilitator of professional development: (a) anticipating teacher responses to rich mathematical tasks; (b) monitoring teachers’ responses to the tasks during the exploration phase; (c) purposefully selecting teacher work to share in whole group discussions; (d) purposefully sequencing the teacher work that will be discussed; and (e) helping the group make mathematical connections between different teachers’ work to develop powerful mathematical ideas. The classroom influence on these principles is clear, for example, viewing the role of the facilitator in terms of monitoring, selecting and sequencing work, which are all recognisable practices for a teacher.

Elliott et al. (2009) suggest the facilitator of discussion may need to ‘scaffold’ teacher responses (again, the word draws on classroom analogies of teaching mathematics). This suggestion relates to a difficulty in using video raised in a comprehensive review of teacher learning with video (Gaudin and Chalies 2015), which is that if ‘scaffolds’ become too specific there is a danger, which Gaudin and Chalies label as ‘mimicking’ (p. 56). In other words, if, as facilitators, we become too explicit about what we want in discussion, there is a danger of teachers mimicking features of ‘productive’ conversations rather than speaking from their experience in a way that opens up that experience to new insight (a mirror, perhaps, of Brousseau's (1984) didactic tension in teaching mathematics).

Drawing on the work cited above, van Es et al. (2014) derived a set of principles that were not a translation of ones developed in the classroom, but that came out of an analysis of professional development discussion. They analysed discussion in their own video clubs and identified four categories that represented key strategies used by experienced facilitators during high quality discussions (defined as those discussions where there was sustained engagement in making sense of students’ thinking or participants’ own thinking). In this writing, I adopt the same definition of high quality or productive discussion, as a key construct for the research. The four categories are:

- Orienting the group to the video analysis task
- Sustaining an inquiry stance
- Maintaining a focus on the video and the mathematics
- Supporting group collaboration (p. 347)

These will be elaborated in turn, while linking to other relevant studies, as they represent one of the most significant current contributions to thinking about facilitating discussion of video in terms of the moves made by facilitators (used for analysis by Tekkumru-Stein and Stein 2017, and Schueler and Roesken-Winter 2018, for example). The full framework includes ‘moves’ linked to each category above, a definition of each move and an example.

‘Orienting the group to the video analysis task’ largely concerns setting up the context of the video to be seen and launching the subsequent discussion. This aspect of the role provides a framing for the whole discussion. Jaworski (1990) noted how the first response by a teacher in discussion of video can set the tone of all that follows and hence this framing and launching are critical in terms of establishing productive ways of working.

‘Sustaining an inquiry stance’ involves six ‘moves’ of the facilitator, conceived in three pairs: (highlighting and lifting up, pressing and clarifying, offering an explanation and countering). A common theme through these moves is the facilitator modelling to the group of teachers what it means to engage in a ‘critical conversation about teaching and learning’ (2014, p. 347). The importance of modelling is also raised in the work of Arya et al. (2013) who analyse literacy teachers’ video-case discussions. They synthesised three practices involved in productive talk (modelling, scaffolding and co-construction) the first of which links closely to what van Es and colleagues describe as sustaining an inquiry stance.

The third practice (van Es et al. 2014) is ‘maintaining a focus on the video and the mathematics’, which is described in terms of grounding discussion in the artefact under study. Nemirovsky et al. (2005) make a relevant distinction here between a ‘*Grounded Narrative* whose aim is to articulate descriptions of classroom events’ from ‘*Evaluative Discourse*’ which ‘centres on the values, virtues and commitments in play’ (p. 365, italics in original). I often use (in the video club itself, and in this writing) the word ‘interpret’ to point to precisely this kind of evaluative discourse. Nemirovsky et al. conclude, ‘Evaluative Discourse is in our experience, by far, the most prevalent mode used in conversation about videotaped teaching episodes’ (p. 388), a finding which is confirmed elsewhere (e.g. Jaworski 1990; van Es and Sherin 2008). Given the seeming difficulty of engaging in grounded narrative and its importance, it is clear that maintaining focus on the video and the mathematics is both complex and of high significance in terms of the potential for teacher learning, although it should be noted that some ways of working on video would not directly relate such a focus to maintaining a grounded narrative. In a related point, Gaudin and Chalies (2015) raise the issue of the cognitive load of video viewing and the problems, particularly with beginning teachers, in their ‘capacity to identify and interpret classroom events’ (p. 29). Focusing on a grounded narrative, and the detail of events, is one mechanism to lessen this cognitive load.

The fourth part of van Es et al.’s framework is ‘supporting group collaboration’, which involved the moves: standing back, distributing participation and validating participant ideas (p. 348). These moves appear closely related to ones relevant to a classroom and, for instance, validating students’ mathematical ideas.

In analysing the talk in a video club, building on the van Es et al. (2014) research, I will suggest there are practices not currently captured in the framework above that are potentially significant in relation to establishing the grounds or basis for high-quality discussion with teachers. In particular, focusing discussion on the detail of events can mean there is some orientation to the task, that there is a sustained inquiry into evidence from the video, that there is a maintenance of a focus on what is on the video itself and, if this all happens, that there is a sense of group collaboration around a common aim. In other words, there is some sense in which focusing on the details of what takes place in a video cuts across all four of van Es et al.’s (2014) practices. In the next section, I set out the background to the research study reported in this article. I then set out the methodology, before looking at the results.

### Research study background

The video clubs, as conceived in this research, last over a 3-month period with participants meeting on six occasions. The clubs are partly inspired by those run in the USA (e.g. van Es et al. 2014). The data from this article is based on outcomes from a video club that ran between May and July 2015. There were seven participants (all volunteers) who were primary school teachers with between 1 and 16 years of experience and including two teachers in senior leadership positions in their schools. It was free to participate in the club, but it required a commitment to attend and engage in activities between meetings (most importantly, to video record in their own classroom). The number of participants could be up to ten, based on principles of collaborative group-working (Brown and Coles 2011). I framed the video club around an action research text (Altrichter et al. 1993) and asked participants to come to the first meeting having read the first chapter and engaged in an activity (from the book) to help them find, or refine, an issue in their mathematics teaching they wanted to develop or investigate. In other words, I set up the video clubs not with any particular pedagogical focus in mind, but instead with the aim of supporting each participating teacher in developing their own practice in relation to mathematics teaching, i.e. the learning goals were personal to each teacher. I was the facilitator of the club reported on here, and none of the teachers previously knew each other, nor me.

In the first session of the club, we worked on a video clip that is freely available online. In subsequent meetings, participants took video recordings in their own classrooms and brought along a selection they had made (of around 4 min) from that video, for others to watch (I still facilitated the meetings). It might seem surprising that participants were willing to share video recordings of their own teaching in such a context and they did express some misgivings. However, as will be elaborated below, the way of working is one that aims to avoid judgement in the initial stages, perhaps helping make it a safe space in which to share details of one’s teaching practice.

The overall aim of the research project in focus in this article was to learn more about facilitating discussion; it was therefore an obvious decision to record discussions, so that transcripts could be generated and the data from the discussion analysed. The research also had a practical aim, to inform the work of others interested in using video and, for this reason, I decided to video-record the first two meetings of the video club, so that clips demonstrating the way of working could be made available to others (see www.mathsvideoclubs.ac.uk). The other meetings were audio recorded. The fact that these recordings were going to be done was made explicit in the advertising of the club. In line with the University of Bristol’s ethical procedure, at the first meeting, there was a discussion about what participants were actually consenting to and an opportunity for questioning, and an explanation of the process for withdrawing from the research. All the data analysed in this article was sent to participants and their approval was gained for its use. Approval for the research was granted by the Faculty of Social Science Ethics Board.

The way of working in the club was inspired by work at the Open University, described in Jaworski (1990) and also set out, in more detail than here, in Coles (2013a, 2013b). The method makes use of 2 to 4-min clips of video, where the first task for participants is to reconstruct (without interpretation or judgement) what happened, before any move to accounting *for* events (Mason 2002) is allowed (see Coles 2013a). The way of working developed out of Gattegno’s (1965) use of mathematical film, which was adapted by John Mason (personal communication) for use with video, in the context of the Open University’s work with mathematics teachers. Different ways of working on video will have different practices and tasks, to focus teachers on particular elements of any given video but, as stated above, a common feature across many ways of working is the desirability of focusing teachers on evidence and initially avoiding evaluation.

## Designing the research—enactivism as a methodology

The methodology behind the project was enactivist (Reid and Mgombelo 2015; Maturana 1987). Enactivism is a research stance that has become increasingly influential in philosophy (Stewart 2010) and is growing in significance within mathematics education, for example, cited as a research paradigm in the Encyclopedia of Mathematics Education (Lerman 2014). Implications of the enactivist stance for the doing of research were explored in a special issue of *ZDM*, *The International Journal of Mathematics Education* (47, 2). For an enactivist project, research is a form of learning. It is a perspective that is informed by systems thinking (Bateson 1972), phenomenology (Merleau-Ponty 1962) and a radical view of biology (Maturana and Varela 1987), that considers change and relationship as the basis of cognition in all living beings. Using an enactivist methodology is not so much a choice for me, as a description of a way of being as a researcher that informs all that I do. From an enactivist perspective, the context of learning from watching a video is so complex that, in this research, no straightforward causality is being sought. Rather, the aim is to raise awareness of possibilities, as facilitators. It is an implication of the enactivist world-view that we can never ‘make’ another living being act in particular ways, rather we ‘occasion’ opportunities and possibilities, but how someone then acts is always a result of their own history and background. From an enactivist stance, ‘[a]ll doing is knowing and all knowing is doing’ (Maturana and Varela 1987, p. 27). Learning is equated with change and knowing is linked to effective action in a particular context.

### Methods

There were five meetings of the club, all lasting 2 h, so the entire dataset comprises of 10 h of recordings (one further meeting was planned but cancelled in unforeseen circumstances). In meetings 2, 3 and 4, we worked on two video recordings from teachers’ lessons (one after the other). In the first meeting, we watched a clip available online and in the final meeting we saw one teacher’s video and then spent time reflecting on the whole process of participants’ involvement in the club. The meetings took place on the following dates (Table 1).

**Table 1:** The dataset

	Meeting 1 Video recorded	Meeting 2 Video recorded	Meeting 3 Audio recorded	Meeting 4 Audio recorded	Meeting 5	Meeting 6 Audio recorded
Date	22/4/15	6/5/15	20/5/15	1/6/15	17/6/15	1/7/15
Videos watched	(Introduction) ‘Alan’s Infinity’	Teacher N Teacher T	Teacher C Teacher J	Teacher P Teacher D	Meeting cancelled	Teacher G (Feedback)

Given that the data takes the form of recordings of discussion, of relevance here is an enactive approach to studying language (Coles 2015). This approach, which I adopted in the current study, details five mechanisms for the study of language, of which two are particularly relevant: the systematic search for pattern; and, equifinality (p. 241). I conducted an analysis on the entire dataset via a systematic search for pattern, starting with the last piece of data collected, on the principle of equifinality, which is explained below. The principle leads to an approach to analysis that involves the systematic search for all examples of a category, rather than a coding of the whole dataset. There might be several searches through the data, looking for examples of different patterns.

The concept of equifinality is associated with early cybernetic or general systems thinking (von Bertalanffy 1969). In early systems thinking, it was assumed a system not in equilibrium was in danger of collapse, and would resolve itself into an equilibrium state, before being triggered out of it by some other event, only to return again to equilibrium; it was only later discovered that many complex, dynamic systems exist continuously in far-from equilibrium conditions (Juarrero 2002, p. 119). ‘Equifinality’ describes how some stability seeking systems could reach the same equilibrium position from a wide variety of initial conditions.

A finding from my collaboration with Laurinda Brown (e.g. Brown and Coles 2008) is that there can be an identifiable sense of equilibrium in the patterns of communication in a classroom that are achieved year-on-year by experienced teachers, independent of the varying initial conditions within each class of students. There can be a sameness in the process of how talk unfolds in a lesson; each year, the specific details of language would be different, but there would be stability in patterns and connections across years, i.e. the system of communication displayed elements of equifinality. Some credence to this suggestion comes from the fact that equifinality is a concept used in some branches of family therapy to describe how family patterns can become constrained (Stroh Becvar and Becvar 2000).

The insight into equifinality, i.e. the existence of stable metapatterns of interaction that become established over time in a group, leads to a principle of enactivist data analysis. Analysis is cyclical. At the end of any phase of research (in this case, the last meeting of a video club), analysis begins with the final piece of data. On the assumption of equifinality, the final piece of data should exhibit the most stable (meta)patterns. Analysis proceeds therefore by identifying patterns in the final data item, and then tracing these patterns systematically back through the rest of the dataset. The intention in the tracing back is not to tell a story of causality, but rather to trace the emergence of pattern. The cycle of interaction between theory and data continues in loops throughout the life of an enactivist project, i.e. data is looked at and talked about, which informs future data collection.

In this project, in the final meeting of the club, participants were invited to reflect on what they had learnt and anything significant they would take away from having attended meetings. A pattern observable during this conversation was that *every* participant mentioned something related to questioning their own immediate ‘judgement’ of situations, or the difficulty of not interpreting events to fit one’s ideas. While I am not suggesting another researcher would necessarily have noticed this pattern, what is significant for enactivist analysis is that the initial patterns are grounded rather than evaluative (to draw on Nemirovsky et al.’s (2005) language), i.e. the pattern is observable in the data and not based on an evaluation of values or virtues.

A random selection of comments is below (phrases linked to judging are italicised). Teacher names are replaced with (random) initials.

- ‘From that very first session when we watched that video and I think that’s the one thing I’ve picked up most from this club is understanding how *you doctor what you watch unintentionally*’ (Teacher N)
- ‘At the very beginning, I found it so difficult just to *be objective* and I have realised that this is a direct reflection of how I am in the classroom. I listen to children and sometimes *I don*’*t listen* to the question for the question’s sake, I move it on, trying to keep that pace high’ (Teacher T)
- ‘I think often we go and watch other teachers, whether it’s peers, or peer teaching or whether you go to observe. A lot of observations, you kind of go, ‘you should have done that differently, you should have done that like that’. It’s actually been really nice just watching and *talking about what you can see non*-*judgmentally*; this is what they did, this, this and this and not trying to make your own subjective, whatever, so I’ve really appreciated that actually’ (teacher D)
- ‘*Just that judgment, being judged and judging* … After we watched that first [video] … *we were making judgments* … but then that wasn’t really reflection’ (teacher J)

Teacher N reflects on his ‘doctoring’ of what he watches, i.e. showing an awareness of how easy it is to interpret observations; similarly, teacher T talks about his difficulties in being ‘objective’, e.g. focusing on the detail of events on the video and teacher D (a senior leader in his school) makes a link to his own observation of other teachers and being non-judgmental. Teacher J explicitly refers to being judgmental in her observations. Having identified this theme, I worked through the whole dataset and any mention of judgement, or difficulties with interpretation, were transcribed with the aim of uncovering further patterns, in a systematic manner.

A striking feature of the comments reported above (from meeting 6) is the number that refer back to the first meeting. It appears as though a significant shift took place during the first meeting, in relation to the move away from judgement. The first meeting is the starting point for analysis and in the next section the focus is just on that meeting. I report on three transcripts, which are all the instances where a comment from a participant gets interrupted or re-focused by the facilitator, in relation to judging or interpreting. I then worked through the data a second time, looking at evidence of change for particular individuals, i.e. instances where their responses in a similar context appeared different from an earlier time.

## Results—facilitating the move away from judgement

The first meeting of the video club is the focus of this section. I report on three transcripts, which are all the instances where a comment from a participant gets interrupted or re-focused by the facilitator, in relation to judging or interpreting. Before getting to these incidents, I offer a brief outline of the time working on the first video, just in the initial ‘accounts of’ or reconstruction phase (Table 2).

**Table 2:** Time allocation in the first video club meeting

Time	Duration	Activity
00.00–02.15	2 min 15 s	Watch video clip
02.15–8.20	6 min 5 s	Discussion: accounts of recording
8.20–9.50	1 min 30 s	Re-watch start of clip
9.50–14.15	4 min 25 s	Accounts of recording
14.15–15.00	0 min 45 s	Re-watch clip
15.00–16.30	1 min 30 s	Accounts of recording
16.30–17.05	0 min 35 s	Re-watch clip
17.05–18.40	1 min 35 s	Accounts of recording

The three incidents are all taken from the first, 6-min, discussion of the video clip shown. The clip was re-shown a further three times, with shorter and shorter sections viewed, in response to questions and disagreements from the group about what took place. At the end of close to 19 min, I moved the group on to offering an account *for* what they had observed.

I present the three transcripts in the form of a narrative, combining what was said with my own, stimulated-recall of the events. After the transcripts/incidents, I offer an analysis initially based on van Es et al. (2014) and indicating elements of the transcript not captured by this framework.

### Incident 1

In the first meeting, having had some time discussing how the group would operate and hearing what participants had done on the pre-meeting tasks, we moved to watch our first video. No participants were expected to take video recordings of lessons before this meeting and so I chose a video clip from the Video Mosaic database (www.videomosaic.org) called ‘Alan’s Infinity’. The task for students in the video is to answer the question: ‘how many numbers are there between zero and one’. The question is posed by the teacher on the video, the class appear to be students of around aged 9 and, for the duration of the clip, the students are engaged in a whole class discussion. I have used this clip before and am aware it can provoke strong responses (both positive and negative) and so hoped it would be suitable to establish the discipline (Jaworski 1990) of starting work on video with the detail of what took place, without initially straying into interpretation.

I was explicit that the initial task would be to simply say what participants saw on the video. I let the video run, pressed stop and as I was returning to my seat one teacher (P) began talking. The first comment, below, refers to J (another teacher in the group) who had mentioned at the start of the meeting that he was interested in promoting more ‘independence’ in the students he teaches.

[Transcription conventions: //text// indicates overlapping speech; [text] is a transcriber comment; [2] indicates a pause of 2 s; other punctuation has been used to give some sense of phrasing; … indicates some text has been skipped, for ease of reading]

P: I could not stop watching, thinking of you [P looks at J] and your independent children [Alf raises his hand towards P] and unfortunately all

//the children that were not paying attention//

Alf: // So, so, so//

J: // Yeah, yeah//

Alf: That’s an interpretation. So, at this stage, the invitation is to say what you saw, what you observed [1] so [1] how did it begin?

I remember feeling taken aback that P had begun talking before any invitation from me (in which I would usually have re-iterated the task of description and staying with the detail). Following P’s comment, I offer feedback in relation to a previously stated invitation around the kinds of talk wanted at this stage of discussion. The distinction I offer here is that it is impossible to observe ‘not paying attention’. What we might observe is, say, children looking away or playing with items on a desk, or talking—from which it is an ‘interpretation’ that they are not paying attention.

I was anticipating making a move consistent with ‘orienting the group to the video analysis task’ as I sat down, i.e. setting up again and re-emphasising how we would be reconstructing what occurred on the video. Given P started speaking before I began, the closest description of my ‘move’ would be ‘re-directing’ (within ‘maintaining a focus on the video and the mathematics’); however, I also interrupt P (not a move within the framework). I could be seen as ‘highlighting’, but not (as in van Es et al.) pointing towards something noteworthy in the video, but rather the opposite, how P was not following the intended discussion norm. The facilitator moves, although different to those currently characterised in the literature, could fall under van Es et al.’s heading of ‘maintaining a focus on the video and the mathematics’.

### Incident 2

My intervention, in incident 1, did not ensure that conversation thereafter remained within the detail of the events on the video (and nor would I have expected it to). After 3 min, the following interchange occurred (G and J are commenting about a student on the video clip).

G: He said that it would not work if your one whole was 10?

J: Yeah, I think he was talking more on the discrete nature of number, he was thinking about things being discrete

Alf: So, try to avoid interpreting what you think he was saying [Alf laughs] try and stay with [1] so, what did you hear him say?

I recognise being attuned, when the task for the teachers is one of description, to any mention by a teacher of what might be going on in the mind of a student on the video. For me, these are the easiest comments to spot that are ‘interpretations’ and not ‘descriptions’. We cannot observe what a student may or may not be thinking, by way of explanation of what they say. So, when J suggests a student was thinking about the discrete nature of number, I am not surprised to observe myself in the transcript intervening and re-emphasising the focus for this phase on evidence from the video.

As in incident 2, I can be seen re-directing attention back to the video; but, also as in incident 1, I highlight what is ‘wrong’ (not part of the van Es et al. framework) with J’s comment at this stage in the video club protocol (it is an interpretation). My laughter might be seen as an awareness of the social awkwardness of such highlighting in the context. As in incident 1, the result seems to be ‘maintaining a focus on the video and the mathematics’.

### Incident 3

The transcript below follows directly from incident 2 (the last line is repeated; turn numbers are added as this is a longer transcript than the other two).

1. Alf: Try to avoid interpreting what you think he was saying [Alf laughs] try and stay with his [1] so what did you hear him say?
2. J: Something about ten objects.
3. P: There’s ten and you cannot get zillionths if there’s just ten.
4. N: And then something about a dust particle.
5. Alf: Was that in the second clip or the first clip? [The ‘clips’ are a reference to the fact that the video is edited and there is an obvious break after a couple of minutes.]
6. J: Second clip, or dust trucks I thought he said.
7. J: I do not know what I heard.
8. Alf: I heard dust bug.
9. P: I think he said dust bits and then someone misinterpreted it as dust bugs.
10. Alf: I thought I heard someone saying effectively there’ll be some unit lower than a dust particle.
11. P: Someone talked about atoms did not they?
12. J: That was when he said about a really long number line.
13. J: I thought that was interesting because
14. Alf: [Alf interrupts T] That
  // sounds like an interpretation //
15. J: //Interpretation, yeah//
16. Alf: Try and stay with detail, we’ll go on to that in a second. Let us try and see if we can get the chronology, so we have got, and we can go back and look, but we got something from the teacher, possibly a question, we think
17. C: How many numbers
18. Alf: Okay

There are disagreements between participants in the early part of this transcript. In line 4, N says he heard something about ‘dust particles’, in line 6, J suggests ‘dust trucks’, in line 8, I suggest ‘dust bugs’ and in line 9, P offers ‘dust bits’. The divergence of ideas here is within the realm of ‘accounts of’ what took place and I can be seen to be engaging in the discussion alongside others; indeed, from lines 2 to 13 (with the possible exception of line 5), it would be hard to distinguish the facilitator acting in any way differently to the participants. It would even be hard to see evidence of facilitator ‘moves’ here, in the sense of van Es et al. (2014), or at least others in the group would also have to be seen to be making similar moves.

However, in line 14, I intervene in a different way, in this case interrupting T’s contribution, and re-stating (line 16) the task as getting ‘the chronology of things’, i.e. *what* happened *when* during the clip. The facilitator move, as in incident 1, is an interruption and a highlighting and re-directing to ‘try and stay with the detail’. In J’s re-voicing of my comment ‘Interpretation, yeah’ there could be evidence of him beginning to recognise the distinction being made here between interpretations and descriptions.

## Discussion: initial reflections on results

Following the three incidents reported above, there are no others where I notice a judgement (either as facilitator at the time, nor as a researcher, subsequently). The teacher discussion remains at the level of detail and ‘accounts of’, characterised by lines 2 to 12 in incident 3, (with three re-viewings of sections of video) before I shift to the next phase of asking for interpretations and analysis of what was seen. In the remaining meetings of the club, during the initial ‘accounts of’ phase of working with video, I make no interventions to bring discussion back to the detail of events and there is an absence of judgments or interpretations made by the teachers. In other words, there is evidence that a discussion norm (about starting off with description and not interpretation) has been established in the first meeting. After three interventions by the facilitator to flag up when discussion has moved to interpretation, no more are needed. Over 20 years of working with OU methods, there is nothing surprising or unusual about the speed with which this happened. This case points to how a focus on the detail of events *can* become established quickly in a group, with a facilitator prepared to intervene and make the criteria for intervention explicit to the group, so that those criteria can become ones that participants are able to apply to themselves. The facilitator moves in evidence, not captured in the van Es et al. framework but linked to ‘maintaining a focus on the video and the mathematics’, seem to centre around highlighting to the group when discussion strayed away from evidence on the video. Highlighting when something has ‘not’ happened perhaps serves to alert teachers to needing to pay attention to the *kinds* of things they are saying.

In reflecting further on the evidence presented above, I now focus on the two questions that have guided this article, *how can a facilitator focus discussion away from judgement and onto the detail of events*, *in the context of professional development using video*? And, *in what ways does focusing on the detail of events occasion subsequent learning for teachers*?

### The role of meta-communication

Reflecting on the way this first meeting went, there is a paradoxical sounding sense in which the facilitator’s own judgmental interpretation of the ‘kind’ of comment made by teachers supported them in moving away from their own judgmental interpretations of the video. The nature of this apparent paradox, that the facilitator appears to support participants moving away from judgement through the use of judgement, is a phenomenon I have not found reported previously and yet it is a move that, in practice, I have become convinced about in terms of effectiveness. In some contexts, ‘do as I say but not as I do’ can put humans in a bind and, when it occurs in a context where it is not possible to question the instigator, can lead to a ‘double bind’ (Bateson 1972, p. 205) that can be psychologically damaging. The moves reported above potentially do seem paradoxical. Yet the apparent effectiveness with which discussion is focused on the detail of events is in contrast to the difficulties reported in establishing discussion norms (e.g. Sherin and van Es 2009) and hence warrants investigation.

When teachers speak judgmentally or evaluatively in the first phase of video watching, my feedback to them indicates *not* that they are making an error in the sense of choosing the wrong alternative, *rather* they are making an error in the set of alternatives from which they are choosing. In other words, I am not questioning the interest or validity of what they say, but what I feedback to them is that they have made an error in terms of the *kind* of thing they are saying—they have made a choice from the wrong set of alternatives (Bateson 1972). My judgments are at a ‘meta’ level to the communications about the video and so do not conflict with them directly. An analogy would be if I ask, ‘What is 7 × 8?’ and someone replies ‘Red’; the response has been chosen from the wrong set of alternatives. In contrast, an answer ‘Fifty-four’ is the correct *kind* of response and so a different kind of error. As a facilitator, I am only concerned with ensuring responses from teachers are of the correct kind. Once responses are in the realm of ‘accounts of’, I am still alert to disagreements and errors (for instance, about words used on the video) but these can be sorted through re-watching; differences in accounts *of* can generate a strong motivation to re-watch (in incident 3, I soon allow a re-watching of the video clip, to try and sort out what was said, the decision making around when to re-watch a clip is discussed in Coles (2013a)).

I interpret the facilitator moves analysed above as offering an example of how it is possible to both constrain discussion (to ensure its focus on the detail) *and* work with teachers, drawing on their own awareness and interest in relation to a video. The facilitator can constrain discussion at a meta-level, i.e. imposing some rules on the kind of comment allowed, while leaving the actual content of discussion to be led by the mathematics teachers, in which the facilitator can take part as another participant. The facilitator is able to offer what might be seen as an ‘enabling constraint’ via imposing a discussion norm that can occasion a re-working of the experience of viewing a video recording. Although the data above is within a particular way of working with video, the moves identified are potentially relevant to any framework that aims to start off discussion in the detail of events, or that privileges evidential comments (e.g. Nemirovsky et al. 2005; Borko et al. 2011).

### Detail and learning

There is only space to consider in a suggestive manner the overall effectiveness of the video club and the question, *in what ways does focusing on the detail of events occasion subsequent learning for teachers*? Answering this question entailed a re-analysis of the data, this time following the contributions of particular teachers from the first to last meeting and considering when there was evidence of differences in response over time (characteristic of learning from an enactivist stance). One relevant piece of data came from the first meeting, when participants were asked (by me) to move on to the interpretation stage, in relation to the video we had watched.

Alf: Any reflections on what the teacher was doing then or what the students were doing, or any teaching strategies?

P: I thought she was very controlled and very restrained. I talk far too much in my maths lessons I think. She just let them get on with it.

There is a difference in P’s comment here, compared to incident 1, when P initially reacted to the video with the statement: ‘I couldn’t stop watching, thinking of you [P looks at J] and your independent children and unfortunately all the children that weren’t paying attention’. P appears to have shifted in her view of the teacher on the video ‘she was very controlled and very restrained’. However, what is more significant is that, whereas the comment from P in incident 1 is, in keeping with Jaworski’s (1990) insights, unlikely to have led to P learning from the video discussion, in the quotation above, P reflects on her own teaching: ‘I talk far too much in my maths lessons I think’. It is hard to imagine P having got to this kind of depth of reflection on her teaching if she had not been challenged to shift the *kind* of comment she had made at the outset, which was about children seeming like they were not paying attention. However, the content of her view (that the children were not paying attention) was never addressed or challenged directly. Her negative evaluation of the classroom on the video, which was perhaps validated by Teacher J (who, in incident 1, commented ‘Yeah, yeah’) might have easily led to a sense that there was little to be learned from analysis of the observed practices. It appears as though the fact of focusing on the detail of the events has been sufficient to allow the space for new thinking to emerge.

In terms of evaluating overall effectiveness of the club from the teacher’s comments, it would be possible to focus on any of them, and I have chosen here three of the seven participants where the evidence of learning is perhaps clearest. Teacher T’s reflections on the video club (part of which were reported earlier), are suggestive of opening himself up to new ways of being in the classroom:

At the very beginning I found it so difficult just to be objective and I have realised that this is a direct reflection of how I am in the classroom. I listen to children and sometimes I do not listen to the question for the question’s sake, I move it on, trying to keep that pace high. And so, I am constantly making interpretations to guide their learning, sometimes that works well but in other times I can make mistakes and so it’s important I ask for confirmation ‘what do you mean?’ so if my interpretation is correct, fabulous, but if not, I can re-think what I was going to do. (Meeting 6)

It would be possible to interpret T’s comments here as being a reflection, in relation to his own teaching practice, that he has been choosing from the wrong set of actions in his classroom (with a focus on pace) and that he has realised he could focus more attention on what students actually mean. He links his learning to a specific strategy that he now uses in his classroom, which is asking students what they mean.

Each teacher made comments that pointed towards potential re-workings of their own practice and these were frequently linked (as teacher T did) to the discipline of starting discussion in a non-judgmental manner. A second example of this is teacher P (who has some senior leadership responsibilities in school) who commented:

I was saying, this process has helped me, when I come to observe now, because I have stopped now thinking about how I would do it and looked at actually what are the children doing, how are they achieving that and what is the teacher doing to get them to achieve that, instead of thinking, oh that’s nice, I’ll nick that, or I’d do it that way, I have switched it back (Meeting 6)

Here, teacher P makes a link between the process of the video club and changes this has made in how she goes about her observations in school of other teachers. The third example of this connection comes from teacher J who commented, in reflecting on the club:

although it’s still sometimes painful watching yourself, you do, instead of watching yourself trying to plug your whiteboard back in, you start thinking about ‘what are those children actually saying’ and actually I realised I made a judgement, or an interpretation about what [student C] was saying but actually I do not think he did mean that and I was too busy thinking, it’s five past three and did not clarify his thinking. (Meeting 6)

Here, teacher J is reflecting on his own teaching and how, in re-watching a video of his own teaching, he caught himself making an interpretation and a judgement about what a student said, rather than making an attempt to hear what was actually said. It appears as though focusing on the detail of the video and being forced to speak evidentially (as is recommended in many ways of working with video) is linked, by these teachers, to their subsequent learning from the video club. And it is the very distinction between an observation and a judgement or interpretation that appears to have been significant. Although this is one example of a video club, the sense of the power of distinguishing observation from judgement fits my experience of using such a way of working with other groups, over a period of 20 years.

## Conclusion

This article set out to contribute to thinking about the role of the facilitator of discussion of video, in particular, how discussion can be kept focused on the detail of events and what a focus on detail might occasion. Through an analysis of the results of an ESRC funded project in the UK, to establish video clubs for teachers of mathematics, some facilitator moves were uncovered that are not within the van Es et al. (2014) framework and yet which seem to be effective in terms of shifting discussion away from evaluation. These moves are related to the van Es et al. category of ‘maintaining a focus on the video and the mathematics’. The moves are in response to a participating teacher straying from a discussion norm, in this case, the explicit invitation to reconstruct what took place (i.e. the detail of what was said, when it was said and who said it). One facilitator move is to actually interrupt a teacher’s contribution to cut short an evaluation or judgement. There may be cultural sensitivities around interrupting that make this move more or less acceptable. However, it does not appear to be the interruption itself that is significant, but what happens next. The second, linked move, is to highlight to the group (but not, in the sense of van Es and colleagues, something important on the video) what it was about the contribution which meant it was not within the parameter of allowable talk at this time in the discussion. Such a move is a meta-comment, in that it is a comment *about* what a teacher has said, for example ‘that’s an interpretation’. Following the highlighting of the breach of the discussion norm, there is then a re-directing back to the task (e.g. ‘let’s stay with the detail of what took place’). The sequence of moves is therefore: (interrupt), highlight/meta-comment on a breach of the discussion norm, re-direct.

It may appear that the facilitator, in making the ‘highlight/meta-comment on a breach of the discussion norm, re-direct’ sequence of moves, is making an interpretation (and judging a teacher’s comment negatively), while at the same time trying to establish a rule of not interpreting. However, it is clear there is a significant difference in the facilitator move and a teacher’s interpretation of the video. If a teacher comments about the video, for example, ‘he was railroading the kids’, this is a negative judgement and evaluative interpretation of actions seen on the video. If a facilitator interrupts with ‘that’s an interpretation’, this is not denying there is validity in the teacher’s comment but pointing out that comments of that *kind* are not the kinds of comments within the discussion norm of this phase of video work. The facilitator moves are at a meta-level to the teacher comments and indicate the teacher has chosen from the wrong set of alternatives, whereas a teacher’s negative evaluation of the video is indicating their belief that, in the video clip, there was a poor choice from a set of possible actions. There is, then, no paradox in offering an evaluative interpretation (*about* the kind of comment being made) with the intention of stopping the teachers from evaluative interpretation of the video recording itself.

Both Arya et al. (2013) and van Es et al. (2014) draw on the idea of a facilitator modelling the kinds of discourse or social and discussion norms desired in a group. Some of the sequences of moves analysed in this article are a different form of modelling, in the sense that they are not moves that others are intended to copy. It is not expected, and would probably not be helpful, that anyone apart from the facilitator engages in commenting *about* the discussion or highlighting what another person has said. What the facilitator does is highlight what is *not* within the norm, while leaving it up to participants to work on what *is* within the norm; and, expressed in this way, it seems clear that the problems of mimicking raised by Gaudin and Chalies (2015) are unlikely to occur. The facilitator points out a distinction (‘that’s an interpretation’) and invites participants to notice that distinction as the discussion unfolds, there is nothing to mimic here, except the making of this vital distinction.

On the contrary, when discussion is within the realms of ‘accounts of’ phenomena, the facilitator contributes to the discussion alongside the other participants and does not appear to take any privileged position. The facilitator participating in such a manner could be viewed as modelling desired forms of interaction. In contrast to studies suggesting discussion norms are hard to establish, three interventions from the facilitator were sufficient to set up, for the rest of the working of the video club, that discussion of video begins in a grounded narrative, focused on the detail of events. This finding is potentially of interest, given that a commonality across several frameworks for using video is a focus on the fine detail of events, as part of the way of working. The meta-focus of the facilitator on the kind of discussion taking place (labelled a ‘heightened listening’ in Coles (2014)) offers an example of how it is possible to both direct and constrain discussion and yet follow the interests and concerns of participants. The facilitator can take responsibility for ensuring discussion is of the kind envisaged in the video club, leaving the content to be the responsibility of the participants (which will also include the facilitator). Of course, it would also be possible for a facilitator to impose constraints on the content of discussion, for particular purposes at particular times.

There are potential implications here for working with teachers new to facilitating professional development and achieving some kind of fidelity in scaling up professional development. A suggestion from this work would be the significance of working with new facilitators to develop awareness of the *kinds* of discussion valued within a programme, and strategies for bringing discussion back to the intended ‘kind’ of focus when it strays. And a method for doing this, implied by the research, would be a discipline of showing short clips of facilitation and focusing on the detail of events initially before moving to interpret or label. One reflection here is that we have reached a point of self-similarity in the system of students, teachers, teacher leaders, educators of teacher leaders. In other words, if we accept the importance of starting work on video by staying with the details, then the kinds of methods that teacher leaders might use to facilitate the learning of teachers would be precisely those methods used to educate teacher leaders in facilitation, through using video.

Although the evidence of this article comes from a single video club, a commonality across the reflections from participants points to the very distinction between an observation and a judgement or interpretation as having been significant in terms of them re-looking at their own practice (both in the classroom and in working with other teachers). There is some suggestion here of one of the reasons why a focus on the detail of events in a video might be important, in that it forces the enactment, on the part of teachers, of the distinction between observation/interpretation. It is an awareness of the fact that we ‘doctor what [we] watch’ (in the words of teacher N) that perhaps occasions the space to open oneself up to other possibilities for interpretation and hence a route to deeper reflection than staying with initial evaluations.

Although the research reported here was from one particular method of using video with teachers, the suggestions around the potential power of teachers working with the distinction between observation and interpretation offer an extra strand of possibilities for other projects and programmes. In other words, across video clubs with different and specific foci, where there is a sense of wanting to work with teacher’s evidential comments on the video, this research suggests that establishing, actively and in discussion, the distinction between observation and interpretation may in itself provide an added benefit and avenue for teacher learning.

## Abbreviations

ESRC: Economic and Social Research Council
OU: Open University
